# Cytomegalovirus Serostatus and Functional Impairment in Liver Transplant Recipients in the Current Era

**DOI:** 10.3390/v13081519

**Published:** 2021-08-01

**Authors:** Nina Singh, Marilyn M. Wagener

**Affiliations:** University of Pittsburgh and Veterans Affairs Pittsburgh Healthcare System, Pittsburgh, PA 15240, USA; mmw5@pitt.edu

**Keywords:** cytomegalovirus, transplant, outcomes, functional status

## Abstract

Background: Whether donor (D+) or recipient (R+) cytomegalovirus (CMV) seropositivity is associated with functional impairment in liver transplant recipients is not known. Methods: Patients included adult liver transplant recipients in the Organ Procurement and Transplantation Network database transplanted over a five-year period from 1 January 2014–31 December 2018. Functional status in the database was assessed using Karnofsky performance scale. A logistic regression model that controlled for potential confounders was used to examine the association of CMV serostatus and functional status. Variables significantly associated with functional status (*p* < 0.05) were then used to develop propensity score and propensity score matched analysis was conducted where each patient was compared with a matched-control with the same propensity score. Results: Among 30,267 adult liver transplant recipients, D+ or R+ patients had significantly lower functional status at last follow-up than the D-R- cohort (OR 0.88, 95% CI 0.80–0.96, *p* = 0.007). In propensity score matched model, D+ or R+ patients had significantly lower functional status than matched-controls (*p* = 0.009). D+ or R+ CMV serostatus (*p* = 0.018) and low functional level (*p* < 0.001) were also independently associated with infections as cause-of-death. Conclusions: D+ or R+ liver transplant recipients had lower functional status and higher risk of deaths due to infections. Future studies are warranted to examine the mechanistic basis of these findings in the setting of transplantation.

## 1. Introduction

Cytomegalovirus (CMV) has long been recognized as a major opportunistic pathogen in organ transplant recipients [1,2]. In addition to overt disease, there are indirect effects of CMV which include rejection, graft loss, opportunistic infections, vascular thrombosis, and new onset diabetes that may contribute to the excess deaths [3]. Although the adverse effects of CMV on the outcomes after transplantation have diminished in the current era of antiviral prophylaxis, CMV continues to have a major negative impact on post-transplant outcomes.

Epidemiologic studies in the general population have documented an association between CMV seropositive status and functional impairment, cognitive decline, and frailty that interfere with or limit normal functioning and activities of daily living [4,5]. CMV seropositivity is indicative of latent state of the virus during which subclinical and low-level replication occurs throughout the lifetime of an individual [6]. The persistent antigen exposure and resultant low-grade inflammatory state has the potential to accelerate host cell and tissue damage leading to frailty, progression of aging and ultimately death [6,7]. Comprehensive assessment of immune profiling in a large cohort of healthy aging adults showed that CMV serostatus was among the factors with most impact on immune dysfunction with aging [8].

In kidney transplant recipients, lower functional status and frailty increased the risk of adverse outcomes including delayed graft function, early hospital readmission, cognitive decline, and mortality [9,10,11,12]. Lower functional status has also been associated with higher mortality in simultaneous kidney pancreas recipients [13], liver transplant recipients [14], as well as lung transplant recipient [15,16]. An estimated 25% of liver transplant candidates are deemed frail and frailty conferred a higher risk of wait-list long-term mortality, increased hospitalizations, and depression [17]. However, to our knowledge, there are no studies that have systematically examined the association between CMV serostatus and functional status after liver transplantation in a large cohort of patients. Using the Organ Procurement and Transplantation Network (OPTN) database, we sought to determine if CMV seropositivity in the donor or the recipient was associated with functional impairment in liver transplant recipients.

## 2. Methods

Data for the study were obtained from the OPTN and Standard Transplant Analysis and Research (STAR) database, a national registry that includes data on all organ transplants performed in the US since 1987. The United Network of Organ Sharing (UNOS) under the U.S. Department of Health and Human Services maintains oversight of OPTN. Patients included in this study were liver transplant recipients ≥ 18 years of age undergoing transplantation in the 5-year period between January 2014 and December 2018 (both years inclusive), with at least one year of follow-up and for whom recipient and donor CMV serostatus and functional status post-transplant was available. This time-period was selected so as to be most reflective of contemporary management practices after liver transplantation. Additionally, while functional status was available on 88% of the patients from 2014–2018; functional status availability was less than 50% prior to 2014. Functional status in the OPTN database is evaluated by the Karnofsky performance scale which assesses the ability to perform daily activities and the level of assistance required in doing so [18]. This comprehensive tool measures patient’s functional level on an 11-point scale ranging from a score of 100 (normal functioning) to 0 (dead) in ten-point increments [14,19,20] (Table 1). A score of ≥80 is considered indicative of normal functional status [18]. Institutional review board of the VA Pittsburgh Healthcare System approved the study under exempt category as this is a publicly available de-identified dataset.

### Statistical Analyses

Stata/SE, version 16.1 (Stata Corp, College Station, TX, USA) was used for all statistical analysis. The primary goal of the study was to determine whether CMV seropositive status of the recipient (R+) or the donor (D+) was associated with functional impairment after transplantation. A logistic regression model was used to assess functional status at last follow-up and CMV serostatus as the predictor. To account for potential imbalances in clinical characteristics, severity of illness or other confounders, additional risk factors such as age, underlying liver disease, model of end stage liver disease (MELD) score, comorbidities at the time of transplant and years of follow-up were included in the model. Variables found to be significantly associated with functional status in logistic regression model (*p* < 0.05) were then used to develop a propensity score and propensity score matching analysis was conducted. For each patient, the model identifies a matched-control with similar propensity score and mathematically accounts for any bias in the probability of the outcome, which was normal functioning status, a binary endpoint. The CMV-specific effect in the model was computed by evaluating the average of the difference between the observed outcome in CMV seropositive patient and the projected outcome in the matched-control.

Both CMV serostatus and functional status were evaluated for their association with survival. Kaplan-Meier survival functions were calculated for each of four CMV serostatus groups (D+R+, D+R-, D-R+, D-R-). The log-rank test was used to assess for equality between the four survival functions. Functional status at last follow-up was also evaluated with a logistic model with the end point of all-cause mortality. The model included risk factors that may portend poor outcome in liver transplant recipients such age, MELD score, dialysis, and allograft rejection. An additional analysis was performed for assessment of deaths due to infections. Deaths attributed to infections included items coded as bacterial peritonitis, pneumonia, generalized sepsis, fungal and viral infections in the registry database. Infection as a cause-of-death was examined using a competing-risk survival regression model

## 3. Results

There were 30,267 liver transplant recipients in the OPTN/UNOS database who were transplanted within the protocol-specified period with known donor and recipient CMV serostatus and for whom functional status was available. Of these, 40.1% (12,150) were D+R+, 22.1% (6676) were D-R+, 22.8% (6896) were D+R- and 15.0% (4545) were D-R- (Table 2). All patients had > 1 year of post-transplant follow-up; the median follow-up was 2.2 years and ranged from 1–12 years. The median MELD score was 22 (IQR 14–32); 30.1% (9108) of the patients had MELD ≥ 30 (Table 2). Overall, 16.6% (5012) of the patients required renal replacement therapy, and 4% (1213) were on life support at the time of transplantation (Table 2).

### 3.1. Functional Status

**Figure 1:**
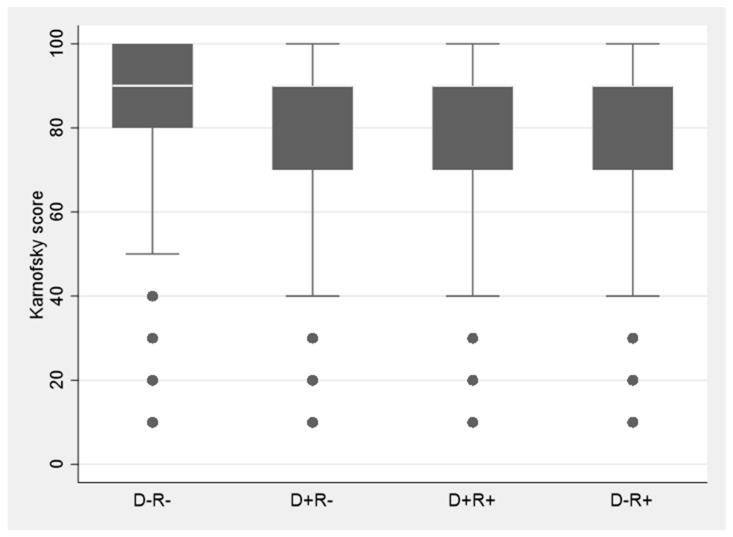
Functional status in each of four donor and recipient CMV serostatus groups. D = donor, R = recipient. CMV D-R- patients had the highest while D+R+ had the lowest functional level. D+R-, D+R+, D-R+ each had significantly lower Karnofsky score than D-R- patients (*p* < 0.005).

In total, 71.9% of the patients at the end of first post-transplant year had normal functioning, 10.7% were capable of self-care but unable to carry on normal activity, 6.6% required occasional or frequent assistance, and 10.8% were disabled (Table 3). Average functional status by CMV serostatus over the study period showed that D-R- cohort had the highest functional level, followed by D-R+, D+R- and D+R+ patients (who had the lowest level of functioning) (Figure 1). D-R+, D+R- and D+R+ groups all had significantly lower functional status than D-R- patients (*p* < 0.005 for each comparison) (Figure 1).

In logistic regression model, D+ or R+ CMV serostatus was independently associated with lower functional status at last follow-up (odds ratio 0.88, 95% CI 0.81–0.95, *p* = 0.001) even when controlled for recipient and donor age, comorbidities at transplantation (MELD, diabetes, need dialysis, need for life support), allograft rejection, and year of transplantation (Table 4). In propensity score matched sample analysis (where each patient was matched with a control with above recipient and donor properties), CMV seropositivity was significantly associated with lower functional level at last follow-up (*p* = 0.009) (Table 5). 

Complete immunosuppression data were available on a subset of patients only (4.6%, 1390/30,267). Of these, 64% received any induction immunosuppression and received 8% T-cell depleting regimens. There was no association between receipt of T-cell depleting induction and functional status; in all 9% (78/872) of the patients with versus 6% (22/341) of those without normal functioning status received T-cell depleting induction (*p* = 0.156). Primary immunosuppressive agent consisted of tacrolimus in 83% of the patients; this subset in propensity score matched analysis also showed a significant decrease in functional status (*p* = 0.001), similar to the full model comprising the entire study population (Table 5).

### 3.2. Mortality

**Figure 2:**
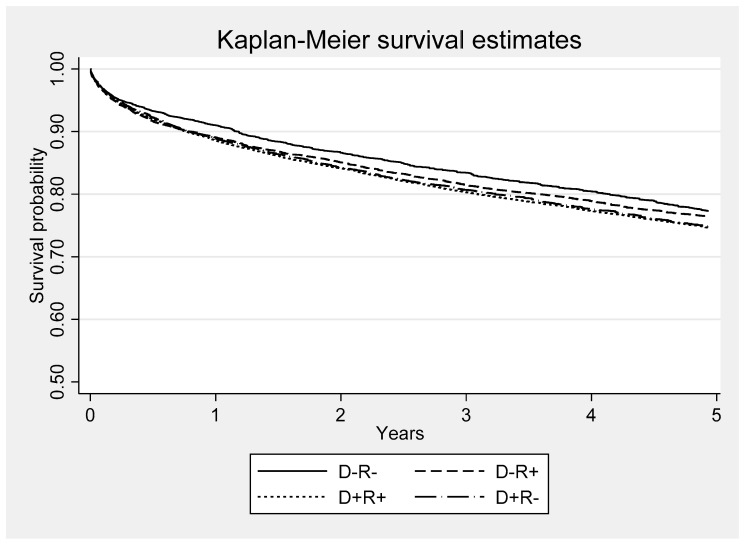
Kaplan-Meier survival functions for each of four donor and recipient CMV serostatus groups. D = donor; R = recipient. All-cause mortality was higher in the CMV seropositive recipient or donor groups when compared to D-R- group. The risk of death was 1.07 (CI 1.03–11) for D-R+, 1.15 (CI 1.11–1.19) for D+R+ and 1.14 (CI 1.10–1.18) for D+R. The four survival curves were significantly different (*p* < 0.001).

All-cause mortality was higher in the CMV seropositive recipient or donor groups when compared the D-R- group. The risk of death was 1.07 (CI 1.03–11) for D-R+, 1.15 (CI 1.11–1.19) for D+R+ and 1.14 (CI 1.10–1.18) for D+R- patients (Figure 2). Overall, the 4 CMV survival curves were significantly different (*p* < 0.001) (Figure 2). Functional impairment was significantly associated with infections as cause-of-death (Table 6). Using the last functional status prior to death, infections were the cause-of-death in < 0.1% (33/21,760) of the patients with normal functional status, 1.0% (33/3228) in those who could perform only self-care, 4.7% (95/2011) in those who required any level of assistance, and 21.1% (690/3268) in patients who were disabled (*p* < 0.001). When controlled for MELD score, recipient and donor age, requirement for dialysis and allograft rejection, D+ or R+ CMV serostatus (*p* = 0.018) was independently associated with greater risk and normal functional level with lower risk (*p* < 0.001) of death due to infections (Table 6). 

## 4. Discussion

Our study shows that CMV seropositivity in the donor or the recipient had a negative impact on functional status in liver transplant recipients. D+ or R+ patients had significantly lower functional status in comparison to their contemporaneous D-R- counterparts. Although the mechanism underlying these associations is not fully understood, growing body of evidence in immunocompetent hosts suggests that latent state CMV (indicative of CMV seropositivity) may have unique role in the development of and/or acceleration of age-related frailty and functional impairment [5,6,7].

CMV latency is characterized by lifelong maintenance of the viral genome in the host tissues. The virus even in its transcriptionally quiescent state continues to express immediate-early genes without progression to the productive forms of the virus [21,22,23]. The ensuing immunostimulatory and inflammatory state triggered by chronic antigenic stimulation over time leads to host cell damage and manifestations of frailty and functional decline [6,7]. CMV antibody levels, considered to reflect multiple CMV reactivations experienced during life, correlated with cognitive decline in older individuals, even when controlled for other risk factors such as age and chronic health conditions [7]. 

Shorter telomere length and reduced telomerase activity have garnered significant interest as potential biomarkers of cellular aging [24,25]. Latent CMV infection has been associated with telomere shortening in the infected cells [24]. Indeed, longitudinal studies of changes in telomere length showed that CMV seropositivity added the equivalent of ~12 years of chronological age in healthy adults 53–76 years of age [26]. These data provide plausibility of our findings about the association of CMV seropositivity with long-term functional impairment. However, the mechanistic basis specifically in the setting of transplantation remains to be elucidated.

Functional impairment also correlated significantly with infections as cause-of death in our study. Higher risk of infections may be explained in part by unique effects of CMV on host immunity with aging. Recurrent antigenic stimulation during CMV latency is characterized by progressive clonal expansion of late-differentiated CD8+ T-cells and a decline in naïve T-cells for recruitment in response to pathogens other than CMV [27,28,29]. Together with reduced repertoire of naïve T-cells due age-related decrease in thymic generation of precursor cells, the ability of the immune system to mount an efficient response against other pathogens and foreign antigens may be compromised rendering the host susceptible to a variety of bacterial and viral infections as well as non-infectious diseases such as atherosclerosis and dementia [30]. While biologically plausible, increased number of committed CD8+ T-cells may not always be detrimental [31] or the sole basis for age-related risk of infections with long-term CMV seropositivity.

There are potential limitations of the study. As with challenges inherent to any registry-based studies, data items may have been missing or misclassified. CMV viremia and disease were not examined in this study as OPTN database does not include these data. However, this does not impact the study findings since the associations reported are in context of CMV serostatus regardless, of whether CMV viremia or disease developed in the patients. Although data on immunosuppressive regimens existed only for a subset of the patients in the registry database, rejection as a surrogate for intensity and net state of immunosuppression was incorporated in all analyses including multivariate and matched-control modeling. Given that Karnofsky performance scale is an observer assessed instrument, determination of functional status may be subject to bias and interobserver variability in general [32] or across different centers in the UNOS database [33] and assessments by health care providers may differ from those by the patients [34]. As such our findings should be considered hypothesis-generating and replicated using measures with greater construct validity and interrater reliability. We also caution that deaths may be multifactorial or unmeasured variables may have had a contributory role.

Strengths of the study are use of a systematically structured and comprehensive database comprising a large sample size that may not be logistically attainable outside the setting of a registry-based platform, with extended and long-term follow-up of the patients. As opposed to single center reports, the data include the entire US transplant population in real-world setting and depict routine clinical practices which enhances generalizability of the findings. Rigorous adjustment for contributory factors and other determinants minimizes their potential confounding effects on the relationship between CMV serostatus and the outcomes examined.

There are wide research and clinical implications of this study. Findings of our study open prospects to examine the immunological and inflammatory mediators by which CMV seropositivity contributes to inferior outcomes many years after transplantation. Identifying and targeting these pathways could pave the way for effective strategies for improving outcomes. The field of immune response modifiers and vaccines for CMV prophylaxis is moving at a fast pace. Whether these agents could provide enduring protection against downstream effects of CMV seropositivity, remains to be determined. Regardless, consideration should be given to assessment of functional outcomes in anti-CMV trials not only at 12 months but also longer-term.

In summary, our study is the first evidence-based demonstration in a large population of liver transplant recipients within the last decade that CMV seropositivity in the donor or recipient was associated with significantly lower functional status at post-transplant and higher risk of deaths due to infections. Future studies are warranted to examine the mechanistic basis of these findings.

## Figures and Tables

**Table 1:** Karnofsky performance scale.

Activity Level	Score	Description
Able to carry on normal activity and to work; no special care needed	100	Normal, no complaints; no evidence of disease
	90	Able to carry on normal activity; minor signs or symptoms of disease
	80	Normal activity with effort; some signs and symptoms of disease
Unable to work; able to live at home and care for most personal needs; varying amount of assistance needed	70	Cares for self; unable to carry on normal activity or do active work
	60	Requires occasional assistance but is able to care for most personal needs
	50	Requires considerable assistance and frequent medical care
Unable to care for self; requires equivalent of institutional or hospital care; disease may be progressing rapidly	40	Disabled; requires special care and assistance
	30	Severely disables; hospital admission is indicated although death not imminent
	20	Very sick; hospital admission necessary; active supportive treatment necessary
	10	Moribund; fatal process progressing rapidly
	0	Dead

**Table 2:** Demographic and clinical characteristics of the 30,267 study patients.

Variable		Number of Patients, *n*/*n* (%)
Demographic Data		
**Recipient**		
Age:	Median (IQR ^1^)	58 (50–64)
	>65 years	6252 (20.7%)
Gender:	Male	19,886 (65.7%)
	Female	10,381 (34.3%)
Race:	White	21,469 (70.9%)
	Hispanic	4293 (14.2%)
	African-American	2772 (9.2%)
	Asian	1249 (4.1%)
	Other/unknown	484 (1.6%)
**Donor**		
Age:	Median (IQR ^1^)	40 (27–53)
	>65 years	2224 (7.4%)
Gender:	Male	18,041 (59.6%)
	Female	12,226 (40.4%)
Cause-of-death: Trauma		8582 (28.4%)
**Medical history**		
Underlying liver disease(s) ^2^:		
Hepatitis C virus		6995 (23.1%)
Alcoholic liver disease		7402 (24.5%)
Non-alcoholic hepatosteatosis		4561 (15.1%)
Cryptogenic		1257 (4.2%)
Hepatocellular carcinoma (any)		8512 (28.1%)
Diabetes mellitus		8516 (28.1%)
Renal replacement therapy at transplant		5012 (16.6%)
MELD ^3^ score, median (IQR)		22 (14–32)
MELD ≥ 30		9108 (30.1%)
On life support		1213 (4.0%)
**Donor and recipient CMV serostatus**		
D+R-		6896 (22.8%)
D+R+		12,150 (40.1%)
D-R+		6676 (22.1%)
D-R-		4545 (15.0%)

^1^ IQR = Interquartile range; ^2^ May have more than one underlying diseases; Table lists top five underlying diseases; ^3^ MELD = Model for end stage liver disease.

**Table 3:** Functional status in study patients by the year of transplant.

Transplant Year (Years of Follow-Up)	Number of Patients at Each Year	Number of Patients with Specified Functional Status			
		Normal Functioning ^1^	Capable of Self-Care ^1^	Requiring Assistance ^1^	Disabled ^1^
2014 (5)	5091	3568 (70.1%)	467 (9.2%)	333 (6.5%)	723 (14.2%)
2015 (4)	5479	3980 (72.6%)	483 (8.8%)	363 (6.6%)	653 (11.9%)
2016 (3)	6304	4546 (72.11%)	640 (10.2%)	400 (6.4%)	718 (11.4%)
2017 (2)	6536	4798 (73.4%)	710 (10.9%)	434 (6.6%)	594 (9.1%)
2018 (1)	6857	4868 (72.6%)	928 (13.5%)	481 (7.0%)	580 (8.5%)

Data are shown for all patients with complete 1-year follow-up. Functional level was assessed by Karnofsky performance score at most recent follow-up. ^1^ Normal function (80–100), capable of selfcare (70), requires assistance (50–60), disabled (10–40).

**Table 4:** Factors associated with normal functional status ^1^ at last follow-up.

Variable	Reference Group	Odds Ratio (95% CI)	*p*-Level
D+ or R+ serostatus	D-R-	0.88 (0.81–0.95)	0.001
Recipient age ≥ 65 years	Recipient age < 65 years	0.87 (0.82–0.94)	<0.001
Donor age ≥ 65 years	Donor age < 65 years	0.80 (0.72–0.88)	<0.001
MELD ≥ 30	MELD < 30	0.94 (0.87–1.01)	0.089
Underlying liver disease			
Hepatitis C virus	Other liver disease	0.86 (0.80–0.91)	<0.001
Alcohol		0.95 (0.89–1.02)	0.172
Nonalcoholic hepato-steatosis		0.89 (0.81–0.98)	0.016
Cryptogenic cirrhosis		0.95 (0.82–1.09)	0.472
Diabetes at transplant	No diabetes	0.78 (0.74–0.83)	<0.001
Dialysis at transplant	No dialysis	0.89 (0.81–0.97)	0.007
Donor cause-of-death (trauma)	Other donor cause-of-death	1.09 (1.02–1.15)	0.006
On life support at transplant	No life support	0.84 (0.72–0.97)	0.020
Any rejection episodes	No rejection	0.76 (0.71–0.83)	<0.001
Year of transplant	2014	1.02 (0.99–1.04)	0.148

^1^ Normal functional status = Karnofsky performance score 80–100.

**Table 5:** Propensity score matched models. Patients with CMV recipient or donor seropositive status donor or recipient were significantly less likely to have normal functional status compared to matched-controls with the same propensity score.

Model ^1^	Average Effect of Donor or Recipient CMV Seropositivity	*p*-Level
Complete dataset	−7.7% (CI −5.8 to −9.6)	0.005
Subset with immunosuppression data available ^1^	−5.6% (CI −3.8 to −7.5)	0.011

Normal functioning was defined as Karnofsky ≥ 80. Variables included in the propensity score model were recipient and donor age, diabetes, dialysis, life support at transplant, trauma as donor cause-of-death, rejection, underlying liver disease and time post-transplant. ^1^ The model includes subset of patients with tacrolimus as maintenance immunosuppressive therapy in addition to the above variables in the model.

**Table 6:** Association of functional status at last follow-up with infection as a cause-of-death in patients who died. Patients with functional impairment were significantly more likely to have infections as cause-of-death compared to those with normal functional status.

Characteristics	Reference Group	Odds Ratio (95% CI)	*p*-Level
D+ ^1^ or R+^2^ CMV ^3^ serostatus	D-R-	1.98 (1.13–3.49)	0.018
Capable of self-care	Normal function	2.09 (0.94–4.63)	0.069
Requires assistance		12.68 (6.55–24.57)	<0.001
Disabled		36.18 (25.96–50.42)	<0.001
MELD ^4^ ≥ 30	MELD < 30	1.24 (0.87–1.76)	0.238
Dialysis at transplant	No dialysis	1.65 (1.10–2.47)	0.015
Recipient age ≥ 65 y	Age < 65 y	1.28 (0.90–1.83)	0.165
Donor age ≥ 65 y	Donor age < 65 y	2.33 (1.63–3.34)	<0.001
Rejection	No rejection	1.65 (1.19–2.28)	0.002

Abbreviations: ^1^ D = donor, ^2^ R = recipient, ^3^ CMV = cytomegalovirus, ^4^ MELD = Model for end stage liver disease.

## Data Availability

The OPTN database is a publicly available database.

## Abbreviations

CMV	Cytomegalovirus
CI	Confidence interval
D	Donor
HCV	Hepatitis C virus
HHS	Department of Health and Human Services
HR	Hazard ratio
NASH	non-alcoholic steatohepatitis
NK	Natural killer cells
MELD	Model for End stage Liver Disease
OPTN	Organ Procurement and Transplantation Network
R	Recipient
RRT	renal replacement therapy
STAR	Standard Transplant Analysis and Research
UNOS	United Network of Organ Sharing
